# *Klebsiella pneumoniae*: Development of Carbapenem Resistance due to Acquisition of *bla*_NDM-1_ During Antimicrobial Therapy in Twin Infants with Pneumonia

**DOI:** 10.3389/fmicb.2015.01399

**Published:** 2015-12-18

**Authors:** Junying Zhu, Baixing Ding, Xiaogang Xu, Demei Zhu, Fan Yang, Hong Zhang, Fupin Hu

**Affiliations:** ^1^Department of Clinical Laboratory, Shanghai Children’s Hospital, Shanghai Jiao Tong UniversityShanghai, China; ^2^Institute of Antibiotics, Huashan Hospital, Fudan UniversityShanghai, China; ^3^Key Laboratory of Clinical Pharmacology of Antibiotics, Ministry of HealthShanghai, China

**Keywords:** *Klebsiella pneumoniae*, NDM-1 metallo-β-lactamase, development of carbapenem resistance, meropenem, *in vivo*

## Abstract

**Objectives:** To identify the mechanism of *in vivo* development of carbapenem resistance in *Klebsiella pneumoniae*.**Methods:** Seven sequential isolates of *K. pneumoniae* were obtained from twin infants with pneumonia. Antimicrobial susceptibility testing was performed by agar dilution method. Carbapenemases including KPC and MβL were initially screened using phenotypic methods, and carbapenemase-encoding genes were identified by polymerase chain reaction and amplicon sequencing. Plasmids of all clinical isolates and the conjugants of resistant isolates were estimated by S1 pulsed-field gel electrophoresis (PFGE). Molecular typing were conducted by PFGE of *Xba*I-digested genomic DNA and multilocus sequence typing.**Results:** For old brother, the first and third isolates were susceptible to meropenem, whereas the second and fourth isolates were resistant (MICs 16 mg/L). The first and second isolates from the young brother were susceptible to meropenem whereas the third isolate was resistant. All the resistant isolates produced NDM-1 metallo-β-lactamase. PFGE of *Xba*I-digested DNA revealed almost identical patterns with similarity indices of above 92% for all the seven isolates. All the isolates had the same sequence type named sequence type 37 (ST37).**Conclusion:** To our knowledge, this is the first documented case of development of carbapenem resistance *in vivo* mediated by NDM-1 metallo-β-lactamase in *K. pneumoniae* during treatment of pneumonia with meropenem.

## Introduction

For the first time in its history, Centers for Disease Control and Prevention [CDCP] (2013) categorized drug-resistant superbugs by threat level. Carbapenem-resistant *Enterobacteriaceae* (CRE) strain makes CDC issue an “urgent threat” in 2012. It is well-known that infections due to extensively drug-resistant CRE are associated with significant morbidity and mortality, and such resistant strains have made their way to the most vulnerable populations, including children (Lledo et al., 2009; Logan, 2012). Production of *Klebsiella pneumoniae* carbapenemase (KPC) or metallo-β-lactamase (MβL) is the main mechanism of carbapenem resistance in *Enterobacteriaceae*. Our previous study reported (Chen et al., 2011) that 70.6% of 109 carbapenem-resistant *K. pneumoniae* isolates produced KPC-2-type carbapenemase, and GIM-1-type or VIM-1-type MβL was detected in 9.2% of the isolates. Very few studies have ever reported development of carbapenem resistance due to *bla*_-KPC_ or losing a porin in *Escherichia coli* or *K. pneumoniae* during therapy (Hong et al., 2005; Elliott et al., 2006). Here, we report the alternate isolation of carbapenem-susceptible and carbapenem-resistant NDM-1 MβL-producing *K. pneumoniae* from twin infants in a children’s hospital and *in vivo* development of carbapenem resistance during carbapenem therapy. We have obtained consent from the legal guardians to publish this case study.

## Materials and Methods

### Bacterial Strains

Seven sequential isolates of *K. pneumoniae* obtained from twin infants in Shanghai Children’s Hospital, Shanghai, China, were isolated from sputum. The strains were identified using VITEK 2 Compact System (bioMérieux, France). *E. coli* ATCC 25922, *Salmonella* ser. Braenderup H9812 and *E. coli* J53 (sodium azide-resistant) were used as quality control strain in antimicrobial susceptibility testing, reference marker in pulsed-field gel electrophoresis (PFGE) and recipient strain in conjugation experiment, respectively. A well-characterized strain of *Citrobacter freundii* 1641 (producing an IMP-9-type metallo-beta-lactamase), *K. pneumoniae* 2528 (producing a KPC-2-type carbapenemase), and *K. pneumoniae* 1789 were used as positive controls for screening carbapenemase genes by polymerase chain reaction (PCR), respectively.

#### Antimicrobial Susceptibility Testing

Antimicrobial susceptibility testing was performed using agar dilution method for determination of minimum inhibitory concentrations (MICs). The results were interpreted according to Clinical Laboratory Standards Institute (CLSI) criteria (Clinical and Laboratory Standards Institute [CLSI], 2014). Carbapenemases including KPC and MβL were initially screened using phenotypic methods such as Modified Hodge Test, and combined disk test with 3′-aminophenylboronic acid (APB) and/or EDTA.

#### Genotypic Detection of β-lactamase Genes

The presence of genes encoding the carbapenemases including KPC, NDM-1, VIM, IMP, SPM, SME, GIM, BIC, SIM, AIM, and DIM were investigated in all the seven clinical isolates by using previously described primers (Qin et al., 2014). PCR amplicons were sequenced and the DNA sequences obtained were compared with those available in the NCBI GenBank database using BLAST searches.

#### Transfer of Carbapenem Resistance

Transferability of carbapenem resistance was analyzed using conjugation experiments in Luria-Bertani broth with sodium-azide resistant *E. coli* J53 as the recipient, as described previously (Zhang et al., 2011). Conjugants were selected on Mueller-Hinton agar supplemented with sodium-azide (150 mg/l) and ertapenem (0.5 mg/l) to select for plasmid-encoded resistance. Plasmids of all clinical isolates and the conjugants of resistant isolates were estimated by S1 PFGE.

#### Clonal Relationships

Clonal relationships were analyzed using PFGE of *Xba*I-digested genomic DNA and multilocus sequence typing (MLST). The results of PFGE were interpreted according to the criteria proposed by Tenover et al. (1995).

#### Clinical History Information

Clinical data were collected via chart review for each patient from the hospital’s unified electronic database. The following parameters were assessed: (1) general demographics, such as age, sex, and other background information; (2) the ward where the patient stayed; (3) use of antibiotics, particularly carbapenems, and extended-spectrum cephalosporins; and (4) potential risk factors for the occurrence and spread of CRKP (Carbapenem-resistant *K. pneumoniae*). Infection or colonization with a CRKP isolate was defined according to CDC’s definition of nosocomial infections (Garner et al., 1988).

## Results

### Antimicrobial Susceptibility Testing

The confirmation of MIC results showed that the first and third isolates from the old brother (Twin A), and the first and second isolates from the young brother (Twin B), were susceptible to carbapenems, whereas the second and fourth isolates from Twin A, and the third isolate from Twin B were resistant to carbapenem (MIC 16 mg/L; **Table 1**). All isolates were susceptible to polymyxin B, tigecycline, amikacin, ciprofloxacin, but were broadly resistant to cephalosporins. As expected, the carbapenem-resistant isolates were resistant to β-lactam/β-lactam inhibitor combinations. The result of phenotypic detection of carbapenemase indicated metallo-β-lactamase production among carbapenem-resistant isolates, and extended-spectrum β-lactamases (ESBLs) production in carbapenem-susceptible isolates, respectively.

**Table 1:** Characteristics of the seven *Klebsiella pneumoniae* isolates.

Isolates	Date	Source	MIC (mg/l)												Carbapenemase	ST
					
			IPM	MEM	ETP	POL	TGC	CAZ	CTX	FEP	AMK	CIP	CSL	TZP		
**Twin A (old brother)**																
A1	2014.4.11	Sputum	0.125	≤0.06	≤0.06	0.5	0.5	16	>128	8	1	≤0.06	8	2		37
A2	2014.4.17	Sputum	16	16	16	0.5	0.5	>128	>128	128	1	≤0.06	>128	>128	NDM-1	37
A2-conjugant			2	2	4	0.25	0.25	>128	>128	16	0.5	≤0.06	128	128	NDM-1	
A3	2014.4.23	Sputum	0.125	≤0.06	≤0.06	0.5	0.5	16	>128	8	1	≤0.06	8	2		37
A4	2014.5.10	Sputum	16	16	16	0.5	0.5	>128	>128	128	1	≤0.06	>128	>128	NDM-1	37
A4-conjugant			2	2	4	0.25	0.25	>128	>128	16	0.5	≤0.06	128	128	NDM-1	
**Twin B (young brother)**																
B1	2014.4.15	Sputum	0.125	≤0.06	≤0.06	0.5	0.5	16	>128	8	1	≤0.06	8	2		37
B2	2014.4.19	Sputum	0.125	≤0.06	≤0.06	0.5	0.5	16	>128	8	1	≤0.06	8	2		37
B3	2014.4.29	Sputum	16	16	16	0.5	0.5	>128	>128	128	1	≤0.06	>128	>128	NDM-1	37
B3-conjugant			2	2	4	0.5	0.25	>128	>128	16	0.5	≤0.06	128	128	NDM-1	


*(1) IPM, imipenem; MEM, meropenem; ETP, ertapenem; POL, polymyxin B; TGC, tigecycline; CAZ, ceftazidime; CTX, cefotaxime; FEP, cefepime; AMK, amikacin; CIP, ciprofloxacin; CSL, cefoperazone/sulbactam; TZP, piperacillin/tazobactam.*

*(2) The MICs of polymyxin B and tigecycline were interpreted following the European Committee on Antimicrobial Susceptibility Testing (EUCAST) criteria (≤2 and ≥4 mg/liter for susceptible and resistant, respectively) and the U.S. Food and Drug Administration criteria (≤2 and ≥8 mg/liter for susceptible and resistant, respectively).*

### Genotypic Detection of β-lactamase Genes

Polymerase chain reaction and DNA sequencing for carbapenemases genes indicated that *bla*_NDM-1_ was present in three carbapenem-resistant isolates. No PCR products were obtained for any of the other carbapenemase genes investigated.

### Transfer of Carbapenem Resistance

**FIGURE 1:**
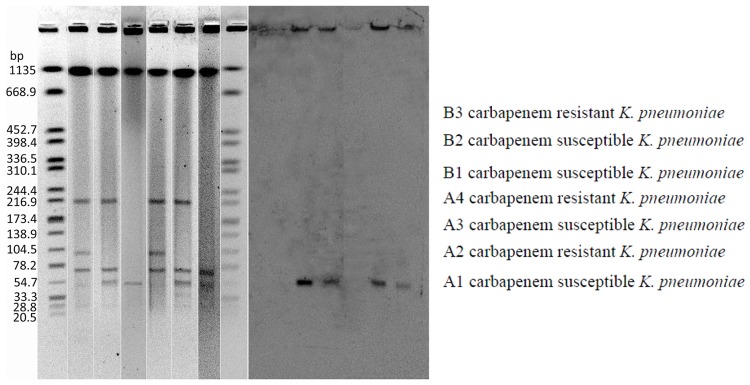
**Plasmid DNAs from *Klebsiella pneumoniae* strains **(A)** and Southern hybridization of plasmid DNAs with a *bla*_-NDM-1_ probe **(B)**.** Lane 1: A1, Lane 2: A2, Lane 3: A2-conjugant, Lane 4: B2, Lane 5: B3, Lane 6: B3-conjugant.

Carbapenem resistance was successfully transferred from all the three carbapenem-resistant *K. pneumoniae* to *E. coli* J53. All conjugants exhibited significantly reduced carbapenem susceptibility to ertapenem (MICs 4 mg/L), and were consistent with detection of *bla*_NDM-1_ in these conjugants. The plasmid containing *bla*_-NDM-1_ is approximately 54 kb (**Figure 1**).

### Clonal Relationships

**FIGURE 2:**
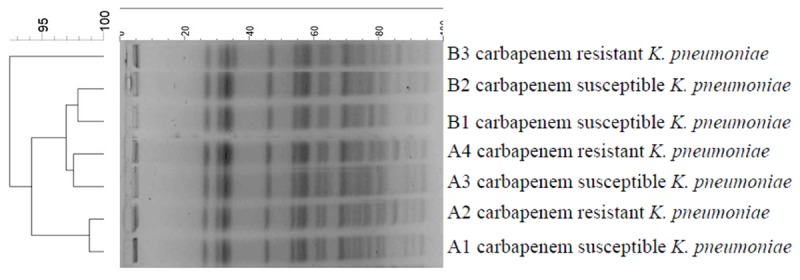
**DNA fingerprinting of *K. pneumoniae* strains by PFGE.** A1, A2, A3, and A4 isolated from twin A (old brother); B1, B2, and B3 isolated from twin B (young brother).

Pulsed-field gel electrophoresis of *Xba*I-digested DNA revealed almost identical patterns with similarity indices of above 92% for all the isolates. However, the PFGE pattern of carbapenem-resistant isolates differed from carbapenem-susceptible isolates by more than one or two bands (**Figure 2**). Likewise, seven isolates had the same sequence type named sequence type 37 (ST37).

### Clinical History Information

The 7-days-old twin infants with acute respiratory distress syndrome were admitted to the ICU in Children’s Hospital of Shanghai. Cefotaxime (0.06 g q12 h) was used prophylactically. (1) For Twin A, pneumonia was diagnosed on admission and mechanical ventilation was provided. A meropenem-susceptible, ESBL-producing *K. pneumoniae* isolate was cultured from sputum specimen 17 days later. The antibiotic therapy was tailored to meropenem 0.09 g per day intravenously, but a meropenem-resistant, *bla*_NDM-1_-positive *K. pneumoniae* isolate was cultured 6 days later. Then the meropenem regimen was changed to meropenem (0.03 g q8 h) plus ampicillin/sulbactam (0.1 g q8 h). About 6 days after discontinuation of meropenem therapy, a third meropenem-susceptible, ESBL-producing *K. pneumoniae* isolate was cultured from sputum specimen. The antibiotic therapy of intravenous meropenem plus ampicillin-sulbactam remained unchanged. The fourth isolate, cultured from a sputum specimen about 17 days later, was found resistant to meropenem. The regimen was modified to meropenem (0.1 g q8 h) plus ampicillin/sulbactam (0.118 g q8 h). No subsequent isolate of *K. pneumoniae* was isolated from sputum, blood, cerebrospinal fluid, central venous catheter tip specimens, or tracheal aspirates any more. Meropenem plus ampicillin/sulbactam was stopped 15 days later. The patient recovered and was discharged from hospital on May 25, 2014. (2) For Twin B, subsequent shock and congenital heart disease were identified firstly. Pneumonia was diagnosed 18 days later. The first and second meropenem-susceptible, ESBL-producing *K. pneumoniae* isolates were cultured from sputum specimen 2 and 4 days later, respectively. The antibiotic therapy was tailored to intravenous meropenem (0.07 g q8 h) plus ampicillin/sulbactam (0.09 g q8 h). After meropenem therapy was discontinued about 10 days later, a meropenem-resistant, NDM-1 MβL-producing *K. pneumoniae* isolate was cultured from a sputum specimen. The patient recovered and was discharged from hospital on May 5, 2014.

## Discussion

The carbapenemase-producing *Enterobacteriaceae* isolates carrying additional resistance genes to multiple antibiotic classes have become resistant to nearly all available therapies. Interestingly, the predominant carbapenemase types of CRE isolates are different between adults and children. For adults, KPC is the primary carbapenemase whereas NDM-1 metallo-β-lactamase is for children (Logan, 2012). Since the first NDM-1 was identified in Kumarasamy et al. (2010) in India, NDM-1-producing *Enterobacteriaceae* have been reported in many countries. In China, the incidence of CRE isolates especially *K. pneumoniae* increased rapidly (Hu et al., 2012). These results suggest that the increasing prevalence of CRE in hospital or environment may attribute to the failure to control the spread of these strains (Walsh et al., 2011). As reported (Gasink et al., 2009; Logan, 2012), the risk factors for acquiring CRE isolates among hospitalized patients included prior use of broad-spectrum antibiotics, mechanical ventilation, indwelling urinary catheters, and change of sickbeds.

Unlike the risk factors of exogenous nosocomial acquired CRE isolate, the endogenous isolate is usually relevant to the use of broad-spectrum antibiotics, especially carbapenems. In this case, resistance emerged unequivocally in the original strain during meropenem therapy, because the findings of PFGE profiles and MLST were almost identical with similarity indices of above 92% for all isolates. Compared with the resistant strains, the susceptible isolates are lack of NDM-1 MβL, which is the major mechanism mediating resistance to meropenem. Curiously, the meropenem-susceptible and –resistant isolates were isolated alternately from the old brother in relation to changing antibiotic regimen. We speculated that the *K. pneumoniae* colonies probably have become heterogeneous population. Either resistant or susceptible variants may become dominant under specific selective pressure of antibiotics.

For the young brother, we cannot exclude the possibility that the final meropenem-resistant, NDM-1 MβL producing *K. pneumoniae* isolate was derived from the initial meropenem-susceptible *K. pneumoniae* by developing carbapenem resistance *in vivo*, but we consider this unlikely, because this final isolate was cultured about 8 days after meropenem therapy was stopped. It is more probable that the final isolate was transferred from the old brother via medical staff, their parents or close contact each other. Another possible explanation is that these *K. pneumoniae* colonies probably exist heterogeneously. Our study also showed that *bla*_-NDM-1_ genes located on plasmid were readily transferable, which implies that the infections caused by carbapenemase-producing *Enterobacteriaceae* isolates might prove difficult to control once they had emerged and rapidly disseminated in clinical setting. Based on our experience, we urge caution on strict infection control measures, including more accurate detection, judicious use of carbapenems, and hand hygiene for containing CRE strains and preventing nosocomial transmission (Lledo et al., 2009; Qin et al., 2014).

To our knowledge, this is the first documented case of carbapenem resistance mediated by NDM-1 metallo-β-lactamase emerging in *K. pneumoniae* during treatment of pneumonia with meropenem. As very few novel antimicrobials have been discovered for the treatment of infections due to CRE isolates, the occurrence of a carbapenem-hydrolyzing enzyme in *Enterobacteriaceae* is disturbing. The spread of CRE and increasing use of carbapenems will make such resistant isolates more prevalent in the world.

## Conflict of Interest Statement

The authors declare that the research was conducted in the absence of any commercial or financial relationships that could be construed as a potential conflict of interest.
